# Antimalarial and Antioxidant Activities of Ethanolic Stem Bark Extract of *Terminalia macroptera* in Swiss Albino Mice Infected with *Plasmodium berghei*

**DOI:** 10.1155/2023/3350293

**Published:** 2023-07-03

**Authors:** Ngouyamsa Nsapkain Aboubakar Sidiki, Noumedem Anangmo Christelle Nadia, Yamssi Cedric, Gamago Nkadeu Guy-Armand, Tientcheu Noutong Jemimah Sandra, Tako Djimefo Alex Kevin, Mounvera Abdel Azizi, Vincent Khan Payne

**Affiliations:** ^1^Department of Animal Biology, Faculty of Science, University of Dschang, P.O. Box 067 Dschang, Cameroon; ^2^Department of Microbiology, Hematology and Immunology, Faculty of Medicine and Pharmaceutical Sciences, University of Dschang, P.O. Box 96 Dschang, Cameroon; ^3^Department of Biomedical Sciences, Faculty of Health Sciences, University of Bamenda, P.O. Box 39 Bambili, Cameroon; ^4^Department of Animal Organisms, Faculty of Science, University of Douala, P.O. Box 24157 Douala, Cameroon

## Abstract

**Background:**

Reduction of oxidative stress during malaria infection is considered as being of great benefit so long as treatment and drug development approaches are concerned. This study had the aim of evaluating the antimalarial and antioxidant activities of the ethanolic extract of *Terminalia macroptera* in Swiss albino mice infected with the *Plasmodium berghei* NK65 strain.

**Methods:**

*In vivo,* the antiplasmodial activity of the plant ethanolic extract was tested in a four-day suppressive and curative assay using *P. berghei* in Swiss albino mice. The extract was administered to the mice at doses of 125, 250, and 500 mg/kg per day. Then, parameters, such as parasite suppression and survival time of the mice, were evaluated. Furthermore, the effect of plant extract on liver damage, oxidative stress indicators, and lipid profile changes in *P. berghei*-infected mice were studied.

**Results:**

Administration of *T. macroptera* significantly suppressed *P. berghei* infection by 55.17%, 70.69%, and 71.10% at doses of 125, 250, and 500 mg/kg, respectively, whereas chloroquine had 84.64% suppression relative to the untreated group 1% Dimethyl sulfoxide (1% DMSO) at day 4 (post-infection) in the four-day suppressive test. This suppression activity rate was dose-dependent. The curative test also presented a significant reduction in parasitemia and an extension of the survival time of the treated groups. Treatment of infected parasitized mice with the extract of *T. macroptera* had a significant (*p* < 0.05) reduction in parameters, such as total protein, aspartate aminotransferase, and alanine aminotransferase. Infection may also lead to a significant increase in the enzymatic activity of liver catalase and superoxide dismutase compared with the normal control group. The non-enzymatic antioxidant activity in parasitized mice was significantly reduced in malondialdehyde and increased in glutathione and nitric oxide when compared with the normal control group.

**Conclusions:**

These findings support the ethnobotanical use of *T. macroptera* stem bark as an antimalarial remedy coupled with antioxidant activity. However, further *in vivo* toxicity tests are required to ascertain its safety.

## 1. Introduction

Malaria is a life-threatening infection caused by a parasite belonging to the genus *Plasmodium*, and five species are traditionally known to cause infection. Malaria is one of the most devastating and prevalent diseases of humans, infecting more than 247 million persons in 2021 in 84 malaria-endemic countries and 600,000 deaths each year with an estimated 95% of deaths in 2020 in African regions [1]. The alarming increase in malaria drug resistance [2] coupled with vector resistance has led to the failure of the prevention and eradication program put in place [3]. Resistance of the parasite to the current artemisinin-based combination therapy [4] has led to the promotion and use of medicinal plants to fight against circulating resistant strains, especially in sub-Saharan Africa [5]. Therefore, scientific investigation of medicinal plants used in herbal medicine to fight against these circulating strains would promote knowledge and a healthy society.

Oxidative stress is a condition, in which dangerous toxic molecules of oxygen and nitrogen intermediates overcome the endogenous antioxidant defense system of the host [6]. According to Nadia et al. [7], there is a close relationship between malaria and oxidative stress. During malaria infection, there is inflammation of the liver, which is the focal point of the parasite life cycle, and this state may lead to production and loss of control of free radicals [8]. However, the production of free radicals in malaria-infected cells can be pathological to the host organism [9]. Antioxidants are substances that neutralize free radicals and stop the harm of reactive oxygen species (ROS) [10]. By neutralizing free radicals before they can damage the cells, antioxidants can significantly lessen the harm caused by oxidants. It is therefore of paramount importance to have an antimalarial drug, which will equally possess antioxidant properties to fight against oxidative stress generated during malaria infection.

*Terminalia macroptera* is a medicinal plant found in most sub-Saharan African countries, such as Cameroon. The stem bark, leaves, and roots of this plant are used in the treatment of malaria by the local population of Noun Division, West Region of Cameroon. Tchatat Tali et al. [11] demonstrated the *in vivo* antiplasmodial activity of *Terminalia mantaly* stem bark aqueous extract in mice infected by *Plasmodium berghei*. Furthermore, the work of Haidara et al. [12] in Mali, evaluated the *in vitro* and *in vivo* antiplasmodial activity of ethanolic extracts of *T. macroptera* leaves and roots on the development of *P. berghei*. However, the *in vivo* antiplasmodial and antioxidant activities of the stem bark of the plant have not yet been evaluated. Therefore, it is of paramount importance to determine the *in vivo* antimalarial and antioxidant activities of the stem bark of *T. macroptera* to scientifically validate its activity. This study was aimed at evaluating the antimalarial and antioxidant activities of stem bark of *T. macroptera* in Swiss albino mice infected with *P. berghei* NK65 to scientifically support its ethnobotanical usage as an antimalarial remedy coupled with its antioxidant activity.

## 2. Materials and Methods

### 2.1. Plant Collection and Identification

The stem bark and leaves of *T. macroptera* were harvested in November 2022 during the dry season from a forest in Noun Division, along the border between the Adamawa and West Regions of Cameroon. A plant taxonomist at the National Herbarium of Cameroon in Yaoundé identified the plant, and a voucher specimen was registered under the number 3053/SRFK. The fresh stem barks of this plant were chopped into small pieces, air dried, and ground into the homogeneous matter with an electric mill and packaged.

### 2.2. Preparation of the Ethanolic Extract of *T. macroptera*

Ethanol extracts were obtained using the procedure described by Josué et al. [13]. Briefly, 100 g of stored powder was macerated in 1.5 L of 95% (v/v) ethanol for 72 hours with occasional stirring. This mixture was filtered using Whatman Paper No. 3. The solvent was evaporated at 45°C in an oven to obtain the extracts. The ethanolic extracts were stored in a refrigerator at 4°C for further usage.

### 2.3. Animal Husbandry and Malaria Parasite

Animals used for the antimalarial studies were bred in the Animal House of the Faculty of Science, University of Yaoundé-Cameroon, weighing between 20 and 30 g. The animals were housed in stainless steel cages with soft wood shavings as bedding, fed a standard commercial pellet meal, provided water ad libitum, and kept in an ambient laboratory environment. The chloroquine (CQ) sensitive strain of *P. berghei* (NK65) was obtained from BEI-Resources, Manassas, VA, USA, and maintained by sub-passage in laboratory mice.

### 2.4. Antimalarial Activity

#### 2.4.1. Peter's Four-Day Suppressive Test

**Figure 1:**
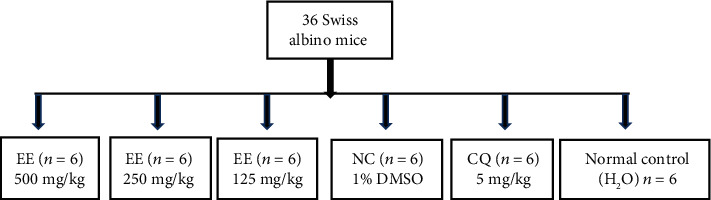
Peter's 4-day suppressive test experimental design.

The method as described by Knight and Peters [14] with slight modifications was adopted. Thirty albino mice were inoculated with 0.2 mL by intraperitoneal injection with standard inoculums of *P. berghei* NK65 with 1 × 10^7^ infected erythrocytes on the first day (day 0). The remaining six mice were not infected (they were given distilled water) and served as normal control. Six *P. berghei*-infected mice were used as untreated negative controls (group 4); group 5 was six *P. berghei*-infected mice treated with 5 mg/kg body weight CQ (positive control); and groups 1, 2, and 3 were six *P. berghei*-infected mice treated with 500, 250, and 125 mg/kg body weight ethanolic extract of *T. macroptera*, respectively. The animals were then randomly divided into six groups of six mice each (Figure 1). Three hours after inoculation, the different groups received treatment. A single dose extract, water, 1% DMSO, and the drug was given daily for 4 days (D0–D3). On the fifth day (D4), slides were labelled, and thin blood films were prepared from the tail of each mouse to evaluate the parasitemia. The average suppression of parasitemia was calculated as follows:

(1) $$\begin{array}{c} \%\text{Parasitemia}=\frac{\left(\text{Number of parasitized RBC}\right)}{\text{Total number of RBCs counted }}\times 100, \end{array}$$

(2) $$\begin{array}{c} \%\text{Suppression}=\left[\left(A-B\right)/A\right]\times 100, \end{array}$$

where *A* is the mean % parasitemia in the negative control group and *B* is the mean % parasitemia in the test group.

#### 2.4.2. Curative or Rane Test

The Deressa et al. [15] approach was used with slight modification. On the first day (D0), 30 mice were intraperitoneally injected with standard inoculums of 1 × 10^7^ parasitized red blood cells (RBCs) from *P. berghei* (NK65). The mice were randomly divided into five groups of six animals each 72 hours later. Thin blood films were first prepared to verify the development of infection. The test groups received a single dose of extract, 1% DMSO, drug, and water as previously indicated above. Tail blood samples from each mouse were collected daily for 4 days, then films were prepared and stained with Giemsa for evaluation of parasitemia. The mean survival rate (MSR) of each group was determined for 30 days as well as the percentage of parasitemia.

(3) $$\begin{array}{c} \text{MSR}=\frac{\text{Number of days survived }}{\text{Total number of days of mice }\left(30\right)}\times 100. \end{array}$$

### 2.5. Antioxidant and Biochemical Parameters Analysis

Three mice per group were sacrificed on the 10th day, and blood and liver samples were collected. Blood was centrifuged at 3000 rpm to obtain serum and stored at 4°C for the evaluation of biochemical parameters, such as alanine aminotransferase (ALT) and aspartate aminotransferase (AST), which were measured using the Dutch Diagnostics kit. The liver tissue was crushed and centrifuged at 3000 rpm for 30 minutes and stored at 4°C for the evaluation of parameters, such as malondialdehyde (MDA) [16], Glutathione (GSH) [17], nitric oxide (NO) [18], protein [18], superoxide dismutase (SOD) [19], and catalase (CAT) [20]. The evaluation of these parameters was done using a spectrophotometer (BIOBASE BK-D560 spectrophotometer). The haematological parameters were equally evaluated using a haematological analyser.

### 2.6. Total Phenolic and Flavonoid Content

Total phenolic and flavonoid content were determined as gallic acid equivalent according to Folin and Ciocalteu [21].

### 2.7. Ethical Approval

All authors hereby declare that the “Principles for the Care of Laboratory Animals” (NIH Publication No. 85-23, revised 1985), such as housing considerations for laboratory animals, bedding considerations for laboratory animals, feeding of laboratory animals, water requirements of laboratory animals have been followed, as well as specific national laws, where applicable [22]. All experiments were reviewed and approved by the Department of Animal Biology, Faculty of Sciences, University of Dschang.

### 2.8. Statistical Analysis

The data generated were analyzed using GraphPad Prism 8.4.2, and the results were expressed as graphs and standard deviation (SD). Each sample was run in triplicates. An Analysis of variance (ANOVA) followed by Tukey's multiple comparisons test was used to compare treated groups with the control group. Values were considered significant at *p* < 0.05. A two-way ANOVA followed by Bonferroni's multiple comparison tests for suppressive and curative tests was employed.

## 3. Results

### 3.1. Antimalarial Suppressive Effects, Parasitemia Level, and MSR

The suppressive effect, parasitemia level, and MSR are all shown in Table 1. It follows from the analysis of Table 1 that CQ and *T. macroptera* extract considerably suppress parasitemia compared with the negative control (1% DMSO). CQ had the highest percent suppression rate of 84.64% compared with 71.10% at 500 mg/kg. Regarding the MSR, there was no significant difference between the extract-treated group and the CQ -treated group.

### 3.2. Curative Test

**Figure 2:**
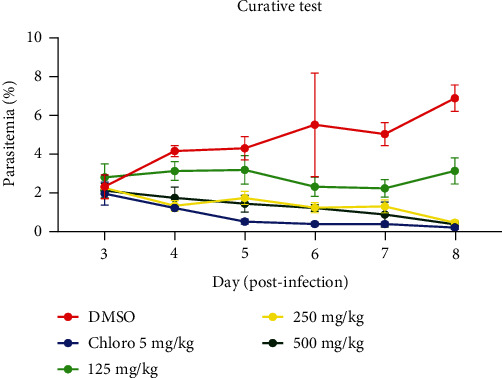
Curative activity of the ethanolic extract of *T. macroptera* stems bark on *P. berghei*-NK65 infected mice.

Figure 2 shows the curative activity of the ethanolic extract of *T. macroptera*. It appears from Figure 2 that the ethanolic extract produced a significant (*p* < 0.05) curative antimalarial effect, especially at doses of 250 and 500 mg/kg starting on days 4–8 compared with the negative control group (1% DMSO). Moreover, the lower dose (125 mg/kg) produced an effect on day 5 and this effect marked a recrudescence of infection on day 7.

#### 3.2.1. MSR of Animals after Treatment with *T. macroptera* during the Curative Test

Table 2 highlights the MSR in mice infected with *P. berghei*. It can be observed from Table 2 that the non-treated group registered the least MSR compared with the treated groups. However, the highest MSR was registered in the group that was treated with CQ and 250 mg/kg.

#### 3.2.2. Haematological Parameters

The effect of ethanolic extract of *T. macroptera* on haematological parameters is shown in Table 3. According to Table 3, the haematological study revealed no significant (*p* > 0.05) differences with the exception of RBCs. However, changes were observed in white blood cell (WBC) count with the greatest reduction in the non-treated group even though not statistically significant (*p* > 0.05). The non-treated group registered reduced values of RBCs with a statistically significant (*p* < 0.05).

#### 3.2.3. Effect of Malaria Infection on Weight and Body Temperature

Table 4 shows the effect of the extract on body temperature during the suppressive test. It appears from the analysis of Table 4, *T. macroptera* extracts prevented body temperature reduction compared with the negative control group. *P. berghei-infected* mice treated with doses 250 and 500 mg/kg exhibited a modest decrease when compared with the negative control; however, this increase was not statistically significant (*p* > 0.05). Furthermore, as compared with the negative control, the extract protected the mice against parasite-induced body weight loss. When the percent change in body weight was evaluated between days 0 and 3, 1% DMSO (*p* < 0.005) and dosage 125 mg/kg (*p* < 0.05) exhibited a significant difference when compared with the treated.

Regarding the curative test, the effect of the extract on the body temperature of *P. berghei*-infected mice is presented in Table 4. Body temperature change measured between days 3 and 7 revealed that plant extract and CQ 5 mg/kg significantly reduced body temperature drop when compared with the negative control. However, when compared with the negative control, this difference was not statistically significant (*p* > 0.005). Furthermore, comparing the % change in body weight between days 3 and 7 revealed that the extract and CQ effectively protected the mice against parasite-induced body weight loss (Table 4).

#### 3.2.4. Biochemical Parameters

Table 5 shows the effect of *T. macroptera* on biochemical parameters. It appears from Table 5 that infection with *P. berghei* showed a considerable rise in the AST, ALT, and protein. Importantly, the serum ALT, AST, and liver protein of untreated, infected mice increased when compared with the non-parasitized non-treated control group, whereas treatment with CQ and *T. macroptera* at different doses (125, 250, and 500 mg/kg) reversed the *P. berghei*-induced alterations in the activity of the biochemical parameters.

#### 3.2.5. Enzymatic Antioxidant Parameter of Infected Animals

**Figure 3:**
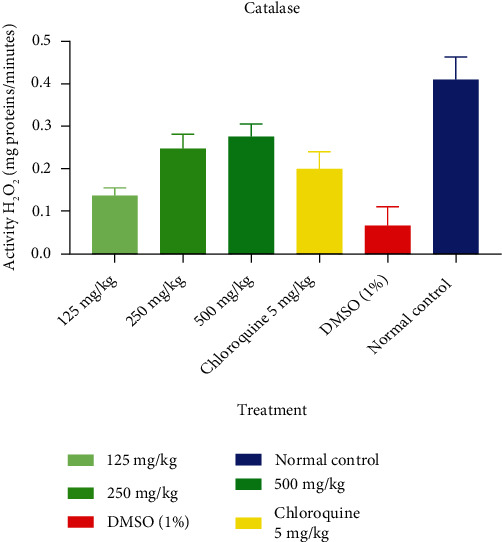
Effect of ethanolic extract of *T. macroptera* on CAT.

Figure 3 shows the effect of *T. macroptera* on CAT. It follows from the analysis of Figure 3 that a depleted antioxidant state was also seen during oxidative stress in the liver *of P. berghei*-infected mice. The parasitized non-treated group (1% DMSO) had lower (*p* < 0.05) liver CAT activity than the normal control, parasitized extract-treated, and CQ (5 mg/kg) groups.

**Figure 4:**
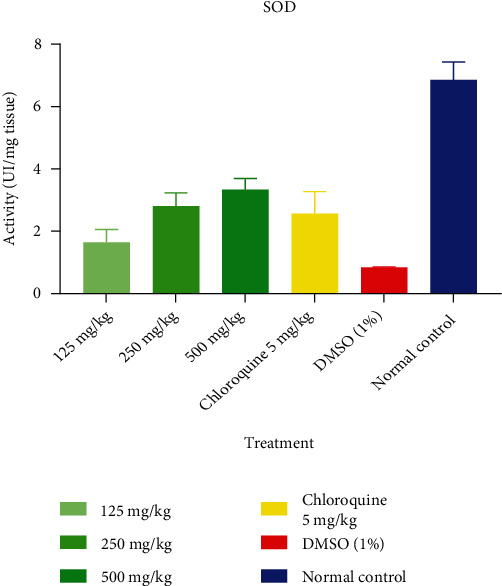
Effect of ethanolic extract of *T. macroptera* on SOD.

Figure 4 shows the effect of the ethanolic extract of *T. macroptera* on SOD. It appears from Figure 4 that the extract-treated groups have a high level of SOD compared with the 1% DMSO-treated group.

#### 3.2.6. Non-Enzymatic Activity

Table 6 displays the effect of *T. macroptera* on glutathione, MDA, and NO. According to the results in Table 6, substantially higher levels of MDA and glutathione (*p* < 0.05) were found in the tissues of the negative control (1% DMSO) than the normal control, and the animals received different dosages. Even though the dosage was unrelated, the administration of various extracts at varied doses resulted in a reduction in tissue MDA and glutathione. Moreover, we can observe a significant increase (*p* < 0.05) in the level of tissue infection caused by a rise in liver NO in the negative control (1% DMSO) group when compared with the normal control. This rise persisted in the group that was not given any treatment, and a minor drop was shown after the administration of the extract in a dose-dependent way. The level of liver NO seems to be slightly normalized after extract administration.

### 3.3. Qualitative Phytochemical Screening

The qualitative phytochemical screening of extracts of *T. macroptera* shows the presence of alkaloids, sterols, triterpenoids, saponins, anthocyanins, and anthraquinones (Table 7).

### 3.4. Total Phenolic and Flavonoid Content

**Figure 5:**
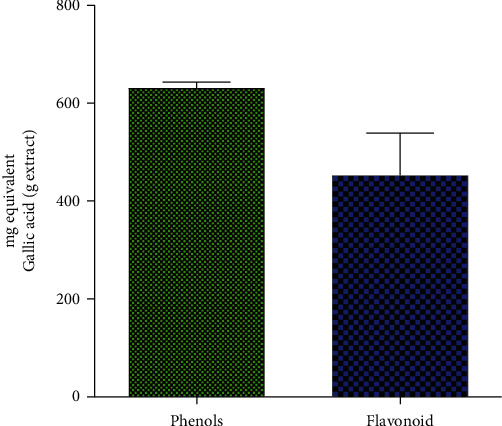
Flavonoid and phenolic contents of ethanolic extracts of *T. macroptera.*

Figure 5 shows the total flavonoid and phenolic contents of *T. macroptera*. In addition, it appears from Figure 5 that the phenolic and flavonoid contents of the ethanolic extract were 631.4 ± 11.5 and 414.4 ± 15.9 mg/g, respectively.

## 4. Discussion

One of the deadliest infectious illnesses in the world is malaria. The main issue endangering all recent advancements in malaria control and having significant effects on public health is the spread and establishment of resistance to first-line antimalarial medications, especially artemisinin [23]. The scientific community is addressing this issue by scouring medicinal plants and other sources for novel, inexpensive, and efficient antimalarial medicines [24]. The present study attempted to investigate the *in vivo* antimalarial and antioxidant potentials of the ethanolic extract of *T. macroptera* in Swiss albino mice infected with *P. berghei* NK65. *T. macroptera* stem bark extract showed an antimalarial activity by suppressing the development of the *P. berghei* parasite by 55.17%, 70.69%, and 71.10% at doses of 125, 250, and 500 mg/kg, respectively. This suggests that the suppressive activity of *T. macroptera* on the growth of *P. berghei* is slightly equal to that of CQ, especially the doses 250 and 500 mg/kg. Moreover, *in vivo,* antimalarial activity can be classified as very good, good, and moderate if the plant extract showed a suppressive rate *X* ≤ 50%, 50% ≤ *X* ≥ 80%, and *X* > 80% at the dose of 125, 250, and 500 mg/kg body weight per day, respectively [25]. According to this classification, the stem bark extract of *T. macroptera* revealed good antimalarial activity. The results obtained are similar to those reported by Omonkhua et al. [26] who observed a suppressive activity of 77, 82% on day 5 with *Terminalia avicennioides* and is in contradiction with the findings of Haidara et al. [12] in Mali, they reported a low chemo-suppression in leaves and roots of *T. macroptera*. The difference observed may be due to the part of the plant used, the method of extraction, the *P. berghei* strain used, and the difference in the geographical location of the plant. The extract equally increases the mean survival time of infected animals. As compared with the negative control, the higher two dosages of the extract substantially or significantly (*p* < 0.05) lengthened the mean survival time of mice. The impact of extending survival time might be directly related to the reduced parasite level in the extract-treated groups. This finding is consistent with previous research by Haidara et al. [12].

In the curative test, even though the ethanolic extract of *T. macroptera* and CQ failed to cure the infection, there was a significant (*p* < 0.05) reduction in the level of parasitemia in a dose-dependent manner during the treatment period, especially at 250 and 500 mg/kg compared with the negative control. Similar observations were done by Omonkhua et al. [26]. However, there was a slight reduction of parasitemia with a dose of 125 mg/kg with a recrudescence of the infection. This phenomenon was also observed by Haidara et al. [12] and could be attributed to the short half-life and a weaker dose used. The curative effect observed could be due in part to the phytochemicals found in the extract, which may have protein-binding and enzyme-inhibiting capabilities, the extract's reported therapeutic effect might indicate that it has a direct effect on the parasites. Furthermore, the MSR of the extract-treated groups was dose-dependent and higher compared with the negative control (1% DMSO), and the group that received the lowest dose, 125 mg/kg, registered a lower survival rate but shorter compared with the standard drug CQ for the curative test. However, the reported mortality in other groups may be a result of the parasite's returns. The findings of this study support those found in Haidara et al. [12]. This finding suggests a very intriguing potential to increase survival time in this treated group with a high mortality rate. However, survival of *P. berghei* infection was related to parasite elimination.

In addition, the ethanolic extract of *T. macroptera* contains phytochemicals, such as alkaloids, triterpenoids, anthocyanins, anthraquinones, and its phenolic and flavonoid contents were 631.4 ± 11.49 and 214.4 ± 15.91 mg/g, respectively. The presence of active substances like alkaloids and flavonoids may be potentially linked to the antiplasmodial action that has been found. Alkaloids have antiplasmodial potential by preventing *Plasmodium falciparum* protein production [27]. According to reports, flavonoids chelate with the parasite's nucleic acid-base pairing [28]. This study is in accordance with previous similar studies [29, 30].

Haematological parameters are of great importance, especially regarding infection with microorganisms (especially malaria parasites). The invasion of the host organism stimulates the immune system leading to the production of WBCs. The adjustment or regulation of these parameters to normal in treated infected groups is an expression of the varied therapies' ameliorative effects. Furthermore, anemia indicators like haemoglobin and RBCs revealed that infection with *P. berghei* induces a considerable drop in these parameters. However, these parameters were regulated to Normal with the administration of CQ and *T. macroptera* extract. This study revealed changes in the RBCs and WBCs in non-treated groups compared with the treated group with similar observations made by Nadia et al. [31] with *Bidens pilosa* where they reported no significant difference in hematological parameters even though slight changes were observed in RBCs and WBCs in the non-treated group. The anemia observed in non-treated groups could be due to the destruction of red blood cells itself caused by the multiplication of the parasite.

One of the most common symptoms of malaria infection is fever, which is usually associated with high temperature. The *P. berghei*-infected mouse model of malaria on the other hand was linked with hypothermia rather than pyrexia. In general, when parasitemia levels rise, mice's body temperature falls. Extracts having active ingredients should help to keep the body temperature from dropping too quickly. In the current study, the extracts protected mice from losing body temperature as compared with the negative control in the tests. This could be due to the presence of an active ingredient found in the plant extract that suppressed or prevent the increase in parasitemia in infected treated mice. Even though a dose of 125 mg/kg caused a drop in parasitemia, it was not sufficient to protect animals of this group from the reduction in body temperature. In addition, an ideal antimalarial medication should equally avoid body weight loss caused by parasitemia. When compared with the negative control (1% DMSO), treatment with ethanolic *T. macroptera* extract resulted in a considerable reduction in body weight loss. This demonstrated that the considerable parasite suppression effect found at the low dosage provided was insufficient to appreciably reduce weight loss. This finding is consistent with prior research on *Dodonea angustifolia* [32] and *Calpurnia aurea* [33]. In contrast, the findings of the prophylactic test indicate that the extract induced a weight increase, which was not observed in previous experiments. This is significantly related to the extract's broad parasite suppression activity, which can prevent parasite-induced body weight loss.

AST is mostly present in the mitochondria of hepatocytes. Because ALT is more specific to the liver, it is a superior measure for identifying liver damage. The ALT and AST activities and serum bilirubin levels are largely used as the most common biochemical markers to evaluate liver injury [32]. Infection with *P. berghei* parasites in mice showed a highly significant increase in serum ALT and AST, equally in liver protein levels compared with the normal control group. The plant extract may have avoided the elevated blood marker enzymes AST and ALT levels. This is consistent with the widely held belief that serum AST and ALT levels revert to normal following hepatic parenchymal repair and hepatocyte regeneration [35]. This may imply that the ethanolic extract of *T. macroptera* may be non-hepatotoxic. Similar observations were made in the study of Oluwatosin et al. [29], where they demonstrated a significant increase in serum ALT, ASAT, and protein in the infected treated group. According to Oh et al. [33], hepatic dysfunction or hepatic damage may be the cause of the elevated serum AST and ALT activities found in the blood of infected mice. Uzuegbu and Emeka [34] observed a rise in the activity of liver damage indicator enzymes. Our findings may indicate that *T. macroptera's* stem bark ethanolic extract may include a hepatoprotective substance and may also be protective against *P. berghei*-induced hepatomegaly in infected mice. According to William et al. [35], the increased biochemical parameters of parasitized non-treated mice were suggested to be due to cellular response to hyper-parasitemia.

CAT converts harmful hydrogen peroxide into water and oxygen and protects the tissues from highly reactive hydroxyl radicals [36]. The changed balance of antioxidant enzymes produced by the reduction in CAT, SOD, and GSH activities may be to blame for the antioxidant defenses' inadequacy in countering ROS-mediated damage. In this experiment, mice treated at doses of 125, 250, and 500 mg/kg *T. macroptera* ethanolic extracts generally showed a significant increase in CAT and SOD activities, and this could be responsible for the cure effect of the extract compared with negative control (1% DMSO). Reduced CAT and SOD activities may be a reaction to increased H_2_O_2_ and O_2_ generation through glucose autoxidation and non-enzymatic glycation . Moreover, the decrease in hepatic SOD and CAT of infected mice observed in this study might be due to the heavy parasite burden, which leads to increased superoxide radical generation. This also suggests that excess ROS probably inactivates these enzymatic antioxidants. This observation is consistent with the findings of Oluwatosin et al. [29], who related lower SOD and CAT activities in *P. berghei* infection to increased ROS production. However, the administration of *T. macroptera* ethanolic extract increased the activities of the antioxidant enzymes. Administration of the extract efficiently lowered SOD and CAT activities to normal, thus protecting the tissue against *P. berghei*-induced oxidative damage.

GSH eliminates free radicals, such as hydrogen peroxide and superoxide radicals, whereas also preserving membrane protein thiols. The most critical mechanism in infection-induced hepatotoxicity is GSH depletion in hepatic mitochondria. In the present study, *T. macroptera* may have a greater ability to reduce oxidative stress by significantly increasing glutathione levels and preventing lipid peroxidation. Furthermore, our findings demonstrated a considerable rise in MDA levels in the livers of infected non-treated groups, which is indicative of lipid peroxidation. These results revealed that treatment of parasitized mice with stem bark extract of *T. macroptera* reduced (*p* < 0.05) elevated liver MDA but increased glutathione to normal when compared with infected non-treated mice. These results are consistent with past observations that described the host's SOD and CAT activities being depleted, and the increase in MDA levels indicating lipid peroxidation in the liver of *P. berghei*-infected mice [37, 38]. The elevated NO level in the tissues of the negative control mice shows that macrophages have an excess of NO to attack *P. berghei*. This rise in NO levels is most likely related to oxidative stress.

## 5. Conclusion

The stem bark extract of *T. macroptera* demonstrated a promising antimalarial activity and contains a compound(s), which may serve as a potential antioxidant source. This plant can, therefore, be a substitute source of a remedy having both antimalarial and antioxidant properties. However, further *in vivo* toxicological studies are necessary to access its safety.

## Figures and Tables

**Table 1:** Suppressive effects, parasitemia level, and MSR.

Treatment	Dosage	Parasitemia level	% Suppression	MSR
Chloroquine	5 mg/kg	4.472 ± 2.982^b^	84.64	27.17 ± 1.641^b^
Ethanolic extract	125 mg/kg	13.29 ± 5.193^b^	55.17	20.33 ± 2.940^b^
	250 mg/kg	8.588 ± 4.758^b^	70.69	26.67 ± 2.472^b^
	500 mg/kg	8.410 ± 5.289^b^	71.10	26.50 ± 1.607^b^
Negative control	1% DMSO	29.10 ± 13.42^a^	—	8.667 ± 1.801^a^

Values are given as mean ± SD (*n* = 3). ^a,b^Values carrying the same superscript letter are not significantly different at *p* < 0.05.

**Table 2:** MSR of the curative test.

Treatment	Dosage	MSR
Chloroquine	5 mg/kg	25.33 ± 3.127^b^
Ethanolic extract	125 mg/kg	18.17 ± 3.177^a^
	250 mg/kg	25.00 ± 1.826^b^
	500 mg/kg	21.67 ± 3.333^b^
Negative control	1% DMSO	7.5 ± 1.384^a^

Values are given as mean ± SD (*n* = 3). ^a,b^Values carrying the same superscript letter are not significantly different at *p* < 0.05.

**Table 3:** Effect of the extract on the hematological parameters in the suppressive test.

		Red blood cells						White blood cells					
Treatment	Dosage	RBC	Hb	HCT (%)	MCV(fl)	MCH	MCCH	WBC	NEU	EOS	BASO	LYM	MON
Negative control	1% DMSO	2.6 ± 0.92^b^	7.3 ± 4.44	15.6 ± 9.924	47.1 ± 4.69	21.9 ± 1.7	47.1 ± 2.5	4.3 ± 0.78	3.6 ± 5.04	0.4 ± 0.38	0.44 ± 0.77	65.61 ± 6.59	29.94 ± 12.77
Chloroquine	5 mg/kg	6.3 ± 0.47^a^	13.5 ± 1.25	29.7 ± 2.3	47.2 ± 0.2	21.3 ± 0.4	45.3 ± 0.8	20.3 ± 12.9	18 ± 6.5	1.38 ± 1.21	1.21 ± 0.24	44.57 ± 4.258	34.7 ± 9.57
Ethanolic extract	125 mg/kg	4.1 ± 1.2^a^	11.5 ± 1.75	23.9 ± 4.86	48.3 ± 2.55	23.4 ± 2.0	48.5 ± 2.9	9.9 ± 1.26	57.3 ± 33.8	0.9 ± 0.73	0.31 ± 0.42	38 ± 26.71	20.8 ± 19.48
	250 mg/kg	5.19 ± 0.76^a^	10.3 ± 1.99	23.9 ± 4.82	45.8 ± 3.05	19.8 ± 1.6	43.6 ± 5.6	20.08 ± 6	8.7 ± 9.16	0.7 ± 0.41	0.7 ± 0.69	35.7 ± 21.02	54.7 ± 24.5
	500 mg/kg	5.8 ± 2.27^a^	11.8 ± 3.89	25.83 ± 9.5	44.9 ± 2.81	20.7 ± 2.1	46.17 ± 1.9	19.09 ± 6.5	5.54 ± 5.6	0.72 ± 0.76	0.47 ± 0.61	45.95 ± 1.104	47.32 ± 6.2
Neutral	Water	6.76 ± 0.54^a^	14.5 ± 0.35	31.70 ± 1.83	47.1 ± 3.5	21.5 ± 1.4	45.7 ± 1.65	11.38 ± 3.3	51.44 ± 39.9	3.6 ± 2.25	0.61 ± 0.49	25.71 ± 21.66	18.64 ± 21.64

Values are given as mean ± SD (*n* = 3). ^a,b^Values carrying the same superscript letter are not significantly different at *p* < 0.05.

RBC: red blood cells; MCV: mean corpuscular volume; HBG: haemoglobin, WBC: white blood cells; HCT: hematocrit; MCH: mean corpuscular content in haemoglobin; MCCH: mean corpuscular concentration in haemoglobin; MO: monocytes; NEU: neutrophils; EOS: eosinophils; BASO: basophils; LY: Lymphocytes; Hb: Haemoglobin.

**Table 4:** Temperature and weight of *P. berghei*-infected mice treated with ethanolic extract of the stem bark of *T. macroptera* in the suppressive and curative test.

	Suppressive test						Curative test					
Treatment	Temperature (°C)			Weight (g)			Temperature (°C)			Weight (g)		
	Day 0	Day 3	%Change	Day 0	Day 3	%Change	Day 3	Day 7	%Change	Day 3	Day 7	%Change
1% DMSO	34.47 ± 1.414	34.08 ± 1.540	−1.14	28.67 ± 2.42^b^	26.67 ± 3.01	−7.23	34.22 ± 1.54	34.08 ± 1.003	−0.41	25.56 ± 6.044	23.56 ± 5.74	−8.14
CQ5 mg/kg	34.72 ± 1.150	34.78 ± 1.270	0.17	24.00 ± 2.09^a^	26.83 ± 3.54	11.14	34.16 ± 1.61	34.42 ± 0.966	0.76	22.44 ± 4.55	24.44 ± 4.24	8.53
125 mg/kg	35.18 ± 0.4262	34.88 ± 0.902	−0.86	27.83 ± 3.76^b^	30.00 ± 3.24	7.50	34.21 ± 1.58	34.47 ± 1.159	0.76	21.67 ± 5.72	24.44 ± 4.24	12.01
250 mg/kg	34.05 ± 1.528	34.53 ± 1.398	1.39	27.33 ± 3.20^a^	28.33 ± 3.50	3.59	33.82 ± 1.20	34.66 ± 1.000	2.45	23.00 ± 3.80	24.67 ± 5.45	7.01
500 mg/kg	35.33 ± 1.011	34.95 ± 1.150	−1.08	26.83 ± 3.25^a^	27.67 ± 3.01	3.08	35.17 ± 1.56	34.94 ± 0.69	−0.66	22.00 ± 4.41	22.67 ± 3.31	2.99
Water	34.23 ± 1.500	34.70 ± 1.092	1.36	22.67 ± 2.33^a^	24.83 ± 2.317	9.09	34.18 ± 1.57	34.31 ± 1.188	0.38	22.22 ± 4.38	23.00 ± 5.38	3.45

Values are given as mean ± SD. ^a,b^Values carrying the same superscript letter are not significantly different at *p* < 0.05.

**Table 5:** Effect of *T. macroptera* on biochemical parameters, such as ALT, AST, and protein.

Group and doses	ALT (serum)	AST (serum)	Protein (liver)
125 mg/kg	18.07 ± 2.329^a^	38.41 ± 3.201^c^	92.08 ± 7.683^b^
250 mg/kg	18.09 ± 0.6305^a^	31.53 ± 3.875^c^	108.7 ± 5.142^a^
500 mg/kg	29.10 ± 7.345^a^	53.51 ± 10.25^a^	123.9 ± 7.920^a^
CQ 5 mg/kg	4.591 ± 2.349^b^	40.16 ± 1.268^c^	103.1 ± 25.74^a^
1% DMSO	53.98 ± 1.310^b^	73.78 ± 3.575^a^	132.7 ± 20.39^b^
Normal control	20.82 ± 5.328^a^	58.78 ± 7.275^a^	88.95 ± 3.487^a^

Values are given as mean ± SD (*n* = 3). ^a,b,c^Values carrying the same superscript letter are not significantly different at *p* < 0.05. ALT: alanine aminotransferase; AST: aspartate aminotransferase.

**Table 6:** Effect of ethanolic extract of *T. macroptera* on glutathione, MDA, and NO parameters.

Treatment	Glutathione (*μ*mol/g tissue)	MDA (*μ*mol/g tissue)	NO
500 mg/kg	1.386 ± 0.1905^a^	0.7318 ± 0.4707^a^	2.355 ± 0.3369^a^
250 mg/kg	1.152 ± 0.1854^b^	0.7212 ± 0.07692^a^	2.078 ± 0.3174^a^
125 mg/kg	1.284 ± 0.08923^a^	0.9562 ± 0.4556^a^	1.714 ± 0.6661^a^
CQ 5 mg/kg	1.055 ± 0.06365^b^	1.381 ± 0.1154^a^	1.778 ± 0.5491^a^
1% DMSO	1.039 ± 0.07671^b^	2.214 ± 0.4424^b^	6.027 ± 1.258^b^
Normal control	1.543 ± 0.06652^a^	0.7874 ± 0.7341^a^	3.169 ± 0.2816^a^

Values are given as mean ± SD (*n* = 3). ^a,b,c^Values carrying the same superscript letter are not significantly different at *p* < 0.05.

**Table 7:** Phytochemical screening of *T. macroptera* ethanolic extracts.

Extract	Alkaloïds	Sterols	Triterpenoids	Saponins	Anthocyanins	Anthraquinones
Ethanolic	+	−	+	−	+	+

+: Positive; −: Negative.

## Data Availability

All data generated and analysed are included in this research article.
